# Digital Peer-Support Platform (7Cups) as an Adjunct Treatment for Women With Postpartum Depression: Feasibility, Acceptability, and Preliminary Efficacy Study

**DOI:** 10.2196/mhealth.9482

**Published:** 2018-02-13

**Authors:** Amit Baumel, Amanda Tinkelman, Nandita Mathur, John M Kane

**Affiliations:** ^1^ Department of Community Mental Health University of Haifa Haifa Israel; ^2^ Psychiatry Research Zucker Hillside Hospital Glen Oaks, NY United States

**Keywords:** mhealth, postpartum depression, perinatal mood disorder, peer support, online, self-help

## Abstract

**Background:**

Peer support is considered to be an important framework of support for mothers experiencing postpartum depression (PPD); however, some barriers exist that may limit its use including peer availability and mothers’ lack of time due to child care.

**Objective:**

This non-randomized study was designed to examine the feasibility, acceptance, and preliminary clinical outcomes of using 7 Cups of Tea (7Cups), a digital platform that delivers self-help tools and 24/7 emotional support delivered by trained volunteers, as an adjunct treatment for mothers diagnosed with PPD.

**Methods:**

Mothers with PPD were referred during intake to the study coach who provided guidance about 7Cups. 7Cups features included self-help tools and chats with trained volunteers who had experienced a perinatal mood disorder in their past. Acceptability was measured by examining self-reports and user engagement with the program. The primary outcome was the Edinburgh Postnatal Depression Scale (EPDS) change score between pre- and postintervention at 2 months, as collected in usual care by clinicians blinded to the study questions. Using a propensity score matching to control for potential confounders, we compared women receiving 7Cups to women receiving treatment as usual (TAU).

**Results:**

Participants (n=19) proactively logged into 7Cups for a median of 12 times and 175 minutes. Program use was mostly through the mobile app (median of mobile use 94%) and between 18:00 and 08:00 when clinicians are unavailable (68% of total program use time). Participants chatted with volunteers for a total of 3064 minutes and have indicated in their responses 0 instances in which they felt unsafe. Intent-to-treat analysis revealed that 7Cups recipients experienced significant decreases in EPDS scores (*P*<.001, Cohen *d*=1.17). No significant difference in EPDS decrease over time was found between 7Cups and TAU, yet the effect size was medium favoring 7Cups (*P*=.05, Cohen *d*=0.58).

**Conclusions:**

This study supports using a computerized method to train lay people, without any in-person guidance or screening, and engage them with patients diagnosed with mental illness as part of usual care. The medium effect size (*d*=0.58) favoring the 7Cups group relative to TAU suggests that 7Cups might enhance treatment outcomes. A fully powered trial has to be conducted to examine this effect.

## Introduction

### Overview

Postpartum depression (PPD) affects 10% to 15% of mothers within the first year after giving birth [1]. While untreated depression and anxiety are associated with low productivity and quality of life [2], PPD carries additional risks due to the baby’s vulnerability to the mother’s state, which may result in a poor mother-child relationship [3] and increased psychopathology in childhood or adolescence [4]. Psychosocial and psychological interventions were shown to be effective treatments for women with PPD [5]; however, some barriers exist that may limit the uptake of evidence-based interventions, including lack of time and child care needs [6]. Technology-based interventions, providing easily accessible services anywhere and anytime needed, could be particularly helpful for these women. In previous studies, it was demonstrated that Internet interventions for mothers with PPD have the potential to significantly reduce depressive symptoms, yielding small to large effect sizes [7-11]. These interventions used mostly principles derived from cognitive behavioral therapy providing users with relevant information, guidelines, and exercises.

Peer support is considered to be another important framework of support for mothers experiencing PPD [12-14]. For example, in a quantitative longitudinal study of 512 first-time mothers, Leahy-Warren et al [15] examined the relationships between social support and PPD and found that at-birth emotional functional support was predictive of PPD at 12 weeks. Websites or virtual communities enabling mothers to easily receive peer support have been studied in the last decade [16-18]. In particular, Evans et al [19] examined the perceived value of women participating in PPD online discussion groups, finding that these groups provide a safe place to connect with others and receive information, encouragement, and hope. Findings suggest that mothers with PPD may find online peer support to be helpful. However, using the Internet as a venue to provide peer support to those who were clinically diagnosed with PPD and as an integral part of mental health care was not fully examined. In addition, technology-based products have yet to demonstrate in real-world settings a scalable way to recruit, train, and engage relevant peers with mothers experiencing PPD.

### Supplementing Treatment With 7Cups, A Digital Platform That Delivers Just-in-Time Peer Support and Self-Help Tools

7 Cups of Tea (7Cups) was chosen for this project because its solution enables, in a scalable way, training users from the community and engaging them with those who seek their support. Another reason to choose 7Cups was the high volume of available volunteers on the platform [20], opening new avenues for providing peer-based emotional support in real-world settings. 7Cups provides free self-help tools and 24/7 emotional support to members through an app or Web-based messaging system. The emotional support is provided by trained volunteers (listeners), who complete a computerized training course on active listening that includes video, text, role-play, and quiz components. A previous study demonstrated that 7Cups users find the listeners’ support to be helpful. Moreover, users found that listeners’ advantages in comparison to psychotherapists lie in their ability to provide sincere care and support without being paid for it (“you got someone who cares enough to listen”) and in listeners’ stand as peers who may better relate to users’ difficulties [21].

Working in collaboration between researchers, clinicians, and patients, we adapted 7Cups to supplement treatment for women with perinatal mood disorders [22]. The first stage enlisted clinicians to identify program modifications necessary to use 7Cups to supplement existing treatment resources. Based on clinician reviews, guidelines for referring patients to use 7Cups were gathered, and a computerized training model was developed to provide listeners with relevant information for supporting women who are coping with perinatal mood disorders. In the second stage, patients with perinatal depression or anxiety used the platform for a single session and provided their evaluation of usefulness and usability and overall impressions of the program. Patients noted a need for support outside the scheduled therapy time and believed that freely available online emotional support could help meet this need. Most patients were interested in receiving support from first-time mothers and those who suffered from perinatal mood disorders in the past [22].

### Study Design

Based on the previous study results, modifications were made, including (1) recruitment of women listeners who had a personal experience of perinatal mood disorders, (2) providing group support tools, (3) integrating relevant evidenced-based self-help tools within the platform, and (4) appropriately presenting relevant information about the listener. Once we were satisfied with the modification process we conducted this pilot study. The primary objectives were to examine (1) whether using 7Cups as an adjunct to treatment is feasible in terms of patient recruitment and timeline (ability to offer the intervention as planned shortly after diagnosis and to assess study outcomes), (2) program acceptability by obtaining participant attitudes toward the use of 7Cups as an adjunct to treatment and examining user behavior on the platform (ie, use patterns), and (3) preliminary efficacy using standardized outcome measures. Our secondary objectives were to explore use patterns in order to understand user preferences and examine whether the experience of receiving lay people’s support felt safe to participants of the study. Patients were referred to use 7Cups in their own environment. We set ourselves the aim of keeping the referral process as close as possible to real-world settings such that patients learned about 7Cups enhancement option as part of their regular intake process.

## Methods

### Participants

Participants were recruited from December 2015 to May 2017 from the perinatal program at the Adult Outpatient Department in the Zucker Hillside Hospital. We aimed to recruit more than 15 participants to enable identify a medium (0.5) pre to post effect size with 90% power and 2-sided 5% significance [23]. Outpatient clinicians, who conducted most of the intakes to the perinatal program, were provided inclusion and exclusion criteria and recruitment material informing participants about the study. Included patients were diagnosed with PPD as indicated by a psychiatrist during intake and recorded in their electronic medical record, 18 years of age or older, treated at the Zucker Hillside Hospital, spoke English, and had home access to the Internet through a computer or mobile phone. Patients were excluded if they had a severe medical disorder, homicidal or suicidal intent or plan, or a history of mental retardation or autism spectrum disorder.

### Procedure

The Feinstein Institute for Medical Research Institutional Review Board approved the study. Patients meeting eligibility criteria who consented to participate came for an in-person meeting with a health technology coach who guided them through the use of 7Cups. Participants completed assessments at baseline and 30 days after. They received up to US $110 compensation for completing assessments, regardless of their use of either 7Cups or usual care.

### Description of 7Cups

**Figure 1:**
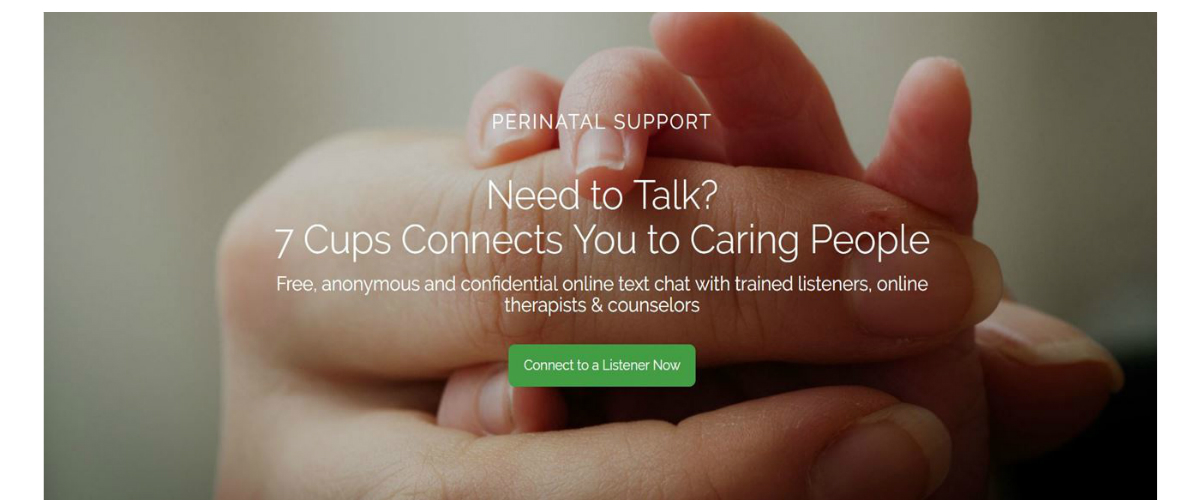
Designated webpage pointing patients to study listeners.

The adaptation of 7Cups as part of the program to supplement treatment for perinatal mood disorders was described in a previous paper [22]. 7Cups provides several components that were introduced to the patients in this study. The first component is the ability to connect with people from the community, mostly listeners. Participants could connect with listeners who opted in specifically to support women with perinatal mood disorders through a designated URL (Figure 1). To be included in this group, listeners had to complete a computerized training course that was developed to provide them with relevant information for supporting women who are coping with perinatal mood disorders [22], and they were required to have a 4.5 average star review based on more than 20 different prior chats. We included only listeners who reported being “past survivors”—that is, to have experienced a perinatal mood disorder themselves.

**Figure 2:**
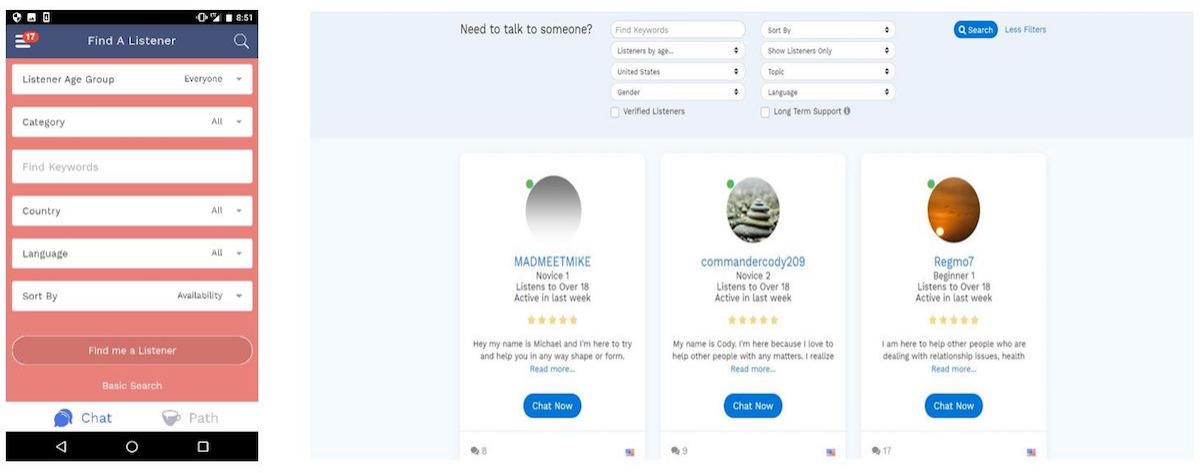
7Cups Browse Listeners feature: mobile (left) and computer (right).

Study participants had the option to browse through the list of general listeners allowing them to filter listeners based on different categories (Figure 2). Participants could also join support groups moderated by listeners. These support groups were accessible 24/7 through open chat rooms that are focused on many topics related to emotional support (eg, anxiety, depression, acceptance and gratitude, disability). Once users chose a support group they would like to join, the chat room would be opened and they could join the conversation.

**Figure 3:**
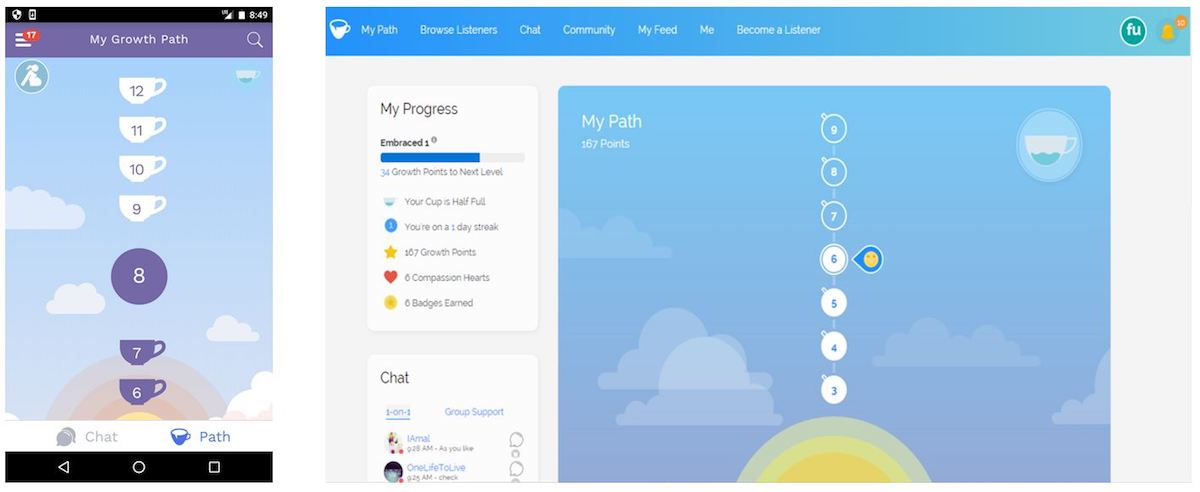
7Cups Growth Path feature: mobile (left) and computer (right).

The second component is a personalized growth path (eg, Perinatal Mood Disorder: Growing Mother; Panic Attacks: Overcoming Panic; Exercise Motivation). Growth paths provide users with a tailored map to help them go from their current state to feeling better (Figure 3). They provide a step-based path to activities or information meant to be therapeutic including gratitude exercises, psychoeducation, exercises drawn from principles of acceptance and commitment therapy, assessments, and feedback. Users can also choose to try something else if they do not like the suggested step. For example, users choosing “Perinatal Mood Disorder” would receive psychoeducational material about how to cope with this disorder as a first step, as a second step would be given an opportunity to try a relaxation exercise, and at the third step would be directed to try one of the support groups embedded within the platform.

The third component is audio-based mindfulness exercises covering topics taken from the mindfulness and relaxation world such as calming meditation and acceptance of thoughts. Enrolled participants could access 7Cups whenever and wherever they chose, through the 7Cups app installed on their device or by computer log-in. The use of 7Cups was completely on demand and patients were not automatically prompted to use it in certain times or situations.

### Health Technology Coaching

Coaching had several aims: installing the app and guiding participants on the program features, tackling any technical difficulties, and encouraging beneficial use through constructive feedback. To enable proper feedback, the coach had access to a platform that presented all user activity in 7Cups. Coaching included 30 minutes in-person guidance (mostly conducted before or after a meeting scheduled for the patient with her clinicians) where users signed in to 7Cups on a computer and then installed it on their mobile device. The health technology coach also proactively contacted each participant 2 to 4 days after enrollment to address any technical difficulties that may have emerged and encourage users to find the features that are most beneficial for them. If, based on program use pattern, patients were not engaged, the coach tried to reach them 1 additional time. Thereafter, participants received 1 to 2 texts within the first 30 days to encourage use and check in on any difficulties that may occur. The study coach had a master’s degree in clinical psychology and did not have any prior clinical experience.

### Assessments and Measures

In this pilot study, we aimed to collect relevant data while keeping the use of the program as close as possible to real-world conditions. Therefore, assessments and analysis were based on 2 paths. First, all participants enrolled into this study received 7Cups as an adjunct to treatment and were measured at preintervention and 30 days after. Second, in order to examine whether 7Cups enhances treatment outcomes, we compared 7Cups users to a different group of patients based on one outcome measure that was available to us. These assessments and analysis are further described in details below.

At baseline, participants completed a demographic questionnaire (age, race, ethnicity, education, employment status, family status, history of mental illness). At baseline and 30 days after enrollment, participants completed online versions of the Beck Depression Inventory II (BDI-II), Edinburgh Postnatal Depression Scale (EPDS), and Beck Anxiety Inventory (BAI). Participants were asked to complete the BAI since symptoms of anxiety might be more common in the perinatal period than in other depressions [24]. EPDS scores are also regularly recorded by psychiatrists during each patient visit for all patients in the perinatal program. We therefore recorded the EPDS score at baseline and 2 months after as collected by physicians or nurse practitioners blinded to the study questions.

Following recommendations of assessing feasibility in applied intervention research [25,26], we evaluated the feasibility of 7Cups as an adjunct treatment based on (1) recruitment (participation) and (2) timeline (ability to offer the intervention as planned shortly after diagnosis and assess study outcomes). Acceptability was measured by examining users’ self-reported attitudes and satisfaction toward using 7Cups as an adjunct to treatment and analyzing program use patterns. Usability (eg, “I find/found 7Cups program easy to use”), attitudes toward 7Cups (eg, “7Cups is/was useful in helping me feel better”), and satisfaction questionnaires (eg, “I would recommend using 7Cups to women who suffer from perinatal mood disorders”) [27-30] that had been adjusted and used in prior studies of 7Cups [21,22,31] were measured at 30 days. 7Cups use data was collected passively. Since the analytical platform did not enable us to effectively differentiate between growth paths use and mindfulness exercises use (which are sometimes also embedded within the growth paths), we examined 7Cups use separated into self-help tools (eg, growth path, mindfulness) and online chat communication (which also included support group use). The length of program use in minutes per session was measured from first log-in until the last event in a given session. When the time between 2 events exceeded more than 5 minutes, a new session was created where the previous session end was the time of the last event recorded in it.

### Data Analysis

Data analysis included descriptive statistics of the multiple-choice questions and program use patterns. Pre-post analysis of self-reported symptoms in BDI-II, EPDS, and BAI were conducted using *t* tests for paired samples.

### Comparison With Treatment as Usual Using a Propensity Score Matching

As described above, the 7Cups enhancement group also received usual care; therefore, it would be difficult to determine whether recorded pre-post effect was only the natural result of usual care. To account for that effect, we created a retrospective comparison group of patients receiving treatment as usual without 7Cups (TAU). As an outcome measure we used the difference in EPDS scores between baseline and 2 months as recorded by the treatment team on a usual basis for all patients in the perinatal program. To control for potential confounders between the groups, several steps were carried out. First, only patients who were not offered 7Cups and met eligibility criteria could be included in TAU group. This condition was possible because different staff members and hospital residents conducted intakes but only some of these “intakers” received guidance and therefore offered patients the use of 7Cups. Second, only patients who had an intake during the study time period could be included in the TAU.

Of these remaining patients, a propensity score matching paradigm with the nearest neighbor matching was used to balance the groups across potential covariates [32,33]. Potential covariates for study outcomes were chosen—EPDS score at intake, diagnosis group (eg, depression, depression and anxiety), age at admission, marital status, race, and ethnicity—and then used to calculate the propensity score for each participant in 7Cups and the TAU prospective sample using SPSS 22.0 (IBM Corp). For each participant in 7Cups, a patient from the TAU prospective sample with the most similar propensity score was selected, without replacement. Balance of the baseline characteristics was assessed postmatching using a measure of standardized bias (similar to an effect size, it is defined as the mean difference divided by the common standard deviation). All standardized biases were acceptable at <0.2 [34,35].

Pre-post analysis of the difference in documented EPDS scores for 7Cups and TAU was conducted using *t* tests for paired samples. Due to the pilot nature of the study and limitations in statistical power, between-group effect size (small 0.20-0.49, medium 0.50-0.79, and large ≥0.80) was also used to compare 7Cups and TAU [36]. Whereas significance testing conveys the likelihood that study results differ from chance expectations, effect-size calculations convey the relative magnitude of the experimental effect and, therefore, provide the opportunity to compare the magnitude of treatment effects within and across studies [37]. We also examined the percentage of participants who experienced a drop in at least 1 level of symptom severity during the 2-month examination based on established severity ranges for EPDS: none or minimal depression (0-6), mild depression (7-13), moderate depression (14-19), and severe depression (19-30) [38].

## Results

### Participants

**Figure 4:**
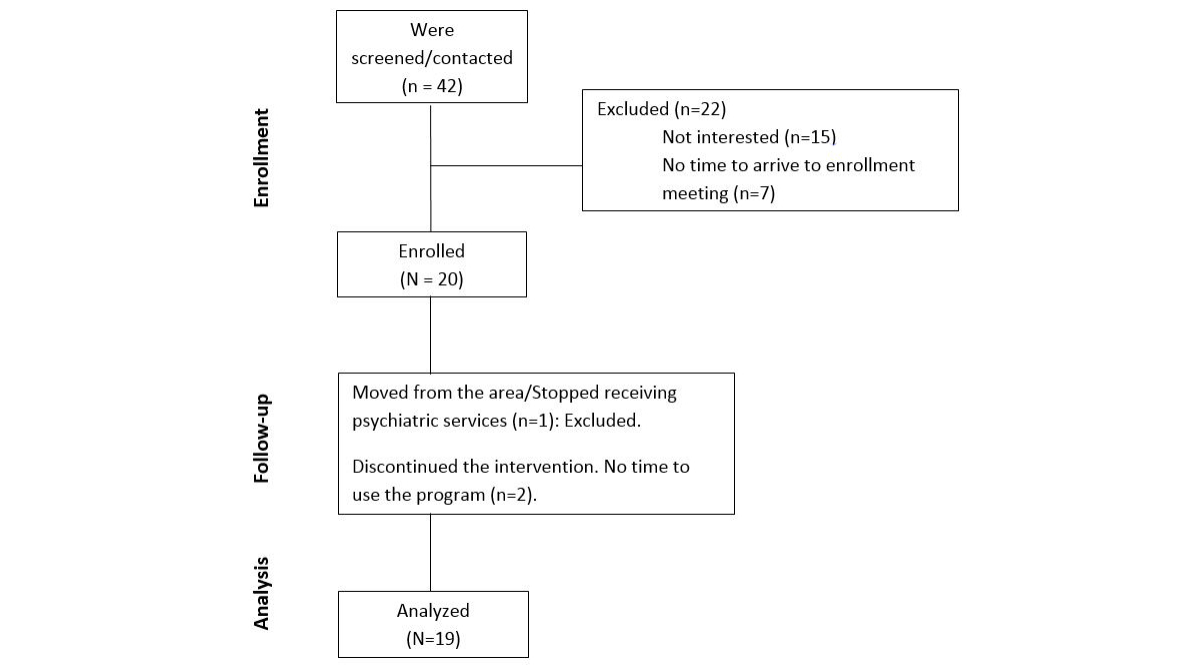
Consolidated Standards of Reporting Trials diagram of participant flow.

A total of 20 patients consented and were enrolled into this study (see Figure 4). Of those patients, 1 participant dropped out due to a move to a different state within the United States and was therefore not included in the analysis. Two participants withdrew from the study voluntarily, however, and were included in the results when applicable based on intent-to-treat analysis. The mean age of participants was 31.95 (SD 5.57) years. All other baseline characteristics of patients can be viewed in Table 1. Of the study sample, 40% (8/20) were black or African American and 15% (3/20) were Hispanic or Latino, most women were married or in conjugal relationships, most women were first-time mothers, and most women had a history of mental illness prior to this treatment period. In terms of usual care, all but 1 participant received antidepressants and most received either individual or group psychotherapy, with a median of 2 in-person meetings within the first 30 days after intake.

### 7Cups Use

**Figure 5:**
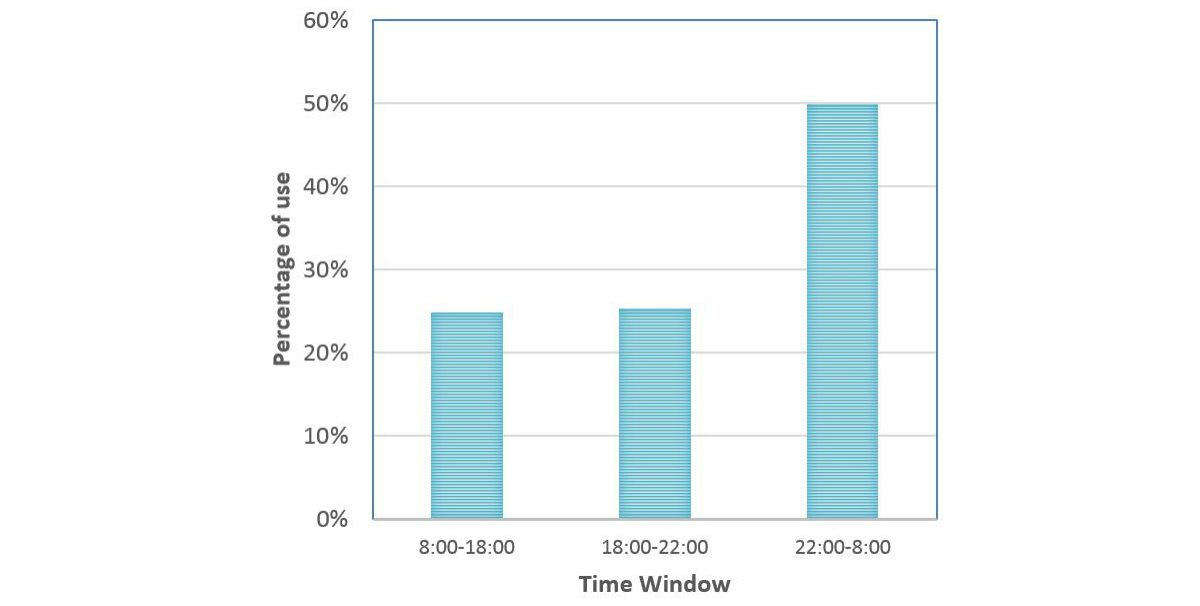
7Cups use by daily time windows.

7Cups use during the 30-day examination can be viewed in Table 2. Participants proactively logged in to 7Cups a median of 12 times and used the program a median of 175 minutes. Participants used 7Cups mostly through mobile devices (median 93.7%). Finally, the median use (in percentages) of listeners’ support was 41.7%. Figure 5 presents the proportion of 7Cups use by daily hours. The data shows that 75% of program use was during evening and nighttime, between 18:00 and 08:00. No significant difference was found between program use in the first 2 weeks and the last 2 weeks of the examination.

### Usability and Acceptability

Participant responses to the usability and acceptability measures are reported in Table 3. A total of 88% (15/17) of study participants found 7Cups to be useful in helping them to feel better, 70% (12/17) indicated that 7Cups emotional support was useful at times when the clinicians were not available, 82% (14/17) indicated they would use 7Cups in the future when needed, and 88% (15/17) indicated they would recommend using 7Cups to women who suffer from perinatal mood disorders. More than 80% (14/17) of study participants indicated they consider 7Cups to be a confidential and safe place. The participants chatted with listeners for an accumulated time of 3064 minutes and indicated in their responses 0 times in which they felt unsafe (ie, feeling any sort of emotional distress or threat caused by listeners’ inappropriate reactions during the chat). One participant wrote she was worried someone from her home might see the data and that a passcode to her mobile phone would be helpful.

### Efficacy

Self-reported outcome measures are presented in Table 4. Paired samples *t* tests indicated significant reductions in symptoms from baseline to post (30 days) in depression on the BDI-II (*P*=.01) and EPDS (*P*=.005). Scores on the BAI did not significantly change. To examine whether there was an association between symptom change and the frequency with which participants used the intervention, we conducted Spearman correlations between changes in BDI-II, EPDS, and BAI scores and the total minutes of use. No significant association was found between these variables.

### Comparison with TAU

This comparison eventually included 17 participants from each group since 2 participants who used 7Cups (2/19, 11%) did not have postintervention clinician-based assessment documented at 2 months after enrollment. These 2 participants did not differ from other participants in their use patterns and self-reports. Intent-to-treat analysis of EPDS scores administered by clinicians revealed that patients in the 7Cups (*t*_16_=4.83, *P*<.001, Cohen *d*=1.17) and TAU (*t*_16_=3.24, *P*=.003, Cohen *d*=0.79) groups experienced significant decreases in depressive symptom severity over the 2-month period. A *t* test for independent samples comparing pre- to posteffect sizes revealed no significant difference between the 7Cups (mean difference 6.29 [SD 5.37]) and TAU (mean difference 3.47 [SD 4.42]) groups, yet the effect size was medium: *t*_32_=1.67, *P*=.05, Cohen *d*=0.58. The percentage of participants who experienced a drop in at least 1 level of symptom severity during the 2-month examination was 71% (12/17) for 7Cups and 47% (8/17) for TAU.

**Table 1:** Sample characteristics (n=20).

Variable		n (%)
**Race and ethnicity**		
	Black or African American	8 (40)
	White	5 (25)
	Hispanic or Latino (white)	3 (15)
	Asian	2 (10)
	Multiracial	2 (10)
**Marital status**		
	Married or in conjugal relationship	16 (80)
	Never married/single	3 (15)
	Divorced/separated	1 (5)
**Highest education**		
	Some high school	1 (5.0)
	Completed high school	1 (5.0)
	Postsecondary school	5 (25)
	Completed 4-year college	7 (35)
	Completed postgraduate training/advance degrees	6 (30)
**Employment status 6 months prior**		
	Employed	14 (70)
	Unemployed	6 (30)
**Number of children**		
	1	11 (55)
	2	7 (35)
	3	1 (5)
	Preferred not to answer	1 (5)
History of mental illness		14 (70)
**Treatment**		
	Psychotherapy or group therapy	17 (85)
	Pharmacotherapy	19 (95)

**Table 2:** Use of 7Cups enhancement during the 30-day examination (n=19).

Use	Median (IQR^a^)	Mean (SD)
7Cups log-ins	12.0 (18.5)	18.0 (16.9)
7Cups use, minutes	175.0 (327.0)	301.7 (412.3)
Proportion of online chat use^b^	41.7 (67.9)	47.0 (36.6)
Proportion of use through mobile	93.7 (18.5)	79.9 (27.2)

^a^IQR: interquartile range.

^b^Calculated based on the time in minutes dedicated to this activity as opposed to self-help. Use of support groups was sparse and accounted for 5% out of the total online chat use.

**Table 3:** Participant responses to usability and acceptability measures (n=17).

Statement	Strongly disagree n (%)	Disagree n (%)	Slightly disagree n (%)	Neutral n (%)	Slightly agree n (%)	Agree n (%)	Strongly agree n (%)
I find/found 7Cups program easy to use	0 (0)	0 (0)	0 (0)	3 (18)	2 (12)	9 (53)	3 (18)
I learned to use 7Cups quickly	0 (0)	0 (0)	0 (0)	4 (24)	3 (18)	6 (35)	4 (24)
7Cups is/was useful in helping me to feel better	0 (0)	0 (0)	0 (0)	2 (12)	1 (6)	13 (77)	1 (6)
The emotional support provided by 7Cups is/was useful in helping me when clinicians weren’t available	0 (0)	1 (6)	0 (0)	4 (24)	2 (12)	5 (29)	5 (29)
7Cups significantly increases/increased the social support I receive/received	0 (0)	2 (12)	0 (0)	3 (18)	3 (18)	8 (47)	1 (6)
I would probably use 7Cups in the future when needed	0 (0)	0 (0)	0 (0)	3 (18)	1 (6)	5 (29)	8 (47)
I would recommend using 7Cups to women who suffer from perinatal mood disorders	0 (0)	0 (0)	0 (0)	2 (12)	0 (0)	6 (35)	9 (53)
I would like to join 7Cups as a listener	1 (6)	2 (12)	0 (0)	7 (41)	1 (6)	2 (12)	4 (24)
I consider 7Cups a safe place	0 (0)	0 (0)	0 (0)	3 (18)	1 (6)	5 (29)	8 (47)
I consider 7Cups a confidential place	0 (0)	0 (0)	0 (0)	3 (18)	1 (6)	5 (29)	8 (47)

**Table 4:** Intent-to-treat (n=19) means and standard deviations of self-reported outcome measures filled by intervention group over a 30-day period.

Measure	Baseline mean (SD)	After 30 days mean (SD)	*t* (pre-post)	*P* value	Pre-post effect size (Cohen *d*)
BDI-II^a^	26.11 (13.34)	19.18 (9.23)	2.48	.01	0.57
EPDS^b^	17.32 (5.96)	13.53 (4.65)	2.88	.005	0.66
BAI^c^	20.47 (13.15)	16.65 (7.52)	1.29	.11	0.30

^a^BDI-II: Beck Depression Inventory II.

^b^EPDS: Edinburgh Postnatal Depression Scale.

^c^BAI: Beck Anxiety Inventory.

## Discussion

### Principal Findings

This study demonstrates that a mobile intervention providing peer support and supplementary self-help tools as an adjunct treatment for women with PPD is feasible and acceptable and suggests that this intervention might also be clinically helpful. Women with PPD used 7Cups more than regular services and mostly when services would not likely be available. Most program use occurred during the evening and nighttime, based on mothers’ (or babies’) schedule. It is also worth noting that while mothers received equivalent guidelines about the use of 7Cups via computer and mobile device, most program use was through mobile device (median of mobile use 93.7%), probably since mobile phones are more accessible when needed. Finally, about a third of mothers who chose not to participate in the study explained they did not have time to attend the enrollment meeting. Overall, these findings are congruent with a previous study showing that for mothers with PPD, one of the main perceived advantages of an online program revolves around its flexibility and accessibility, due to the mothers’ need to manage themselves around the children’s schedules [39].

This study also demonstrated that women with PPD find online peers who have been recruited, trained, and screened using only a computerized program to be helpful and find the experience safe. Participants chatted for a total of 3064 minutes with not one noted incident of feeling unsafe. While a previous study demonstrated that people in emotional distress may find online peer support to be helpful, that study sample was biased as it was composed only of 7Cups native users [21]. Our study provides evidence about the acceptance of a volunteer-based support program from an unbiased sample, which converges with findings from previous studies demonstrating that online peer support can be beneficial for women experiencing depression [19,40]. It is worth noting that the use of support groups was sparse and accounted for 5% out of the total online chat use. It might be that participants would use this feature much more in the presence of an ongoing support group in which the same users attended, as happens in outpatient settings. This, however, raises more complications in terms of product design.

While 7Cups had a very large pre to post positive effect on symptoms of depression (*d*=1.17), the results did not indicate a significant difference between 7Cups enhancement and TAU groups. Since the perinatal program at Zucker Hillside Hospital provides evidence-based care including pharmacologic and psychosocial interventions (eg, psychotherapy, group support), it was expected that TAU would yield significant improvement in symptoms of depression as well. However, the medium effect size (*d*=0.58) favoring 7Cups relative to TAU suggests that 7Cups might improve treatment outcomes. A fully powered trial must be conducted to examine this effect. It is also worth noting that participants did not report a significant drop in symptoms of anxiety, although this may be attributed to lower rates of experienced anxiety (BAI mean 20.47) in comparison to depression (BDI-II mean 26.11) at preintervention.

### Using Technology in the Service of Human Connection

This study presents an intervention that uses a computerized method to train lay people without any in-person guidance or screening and then engages them with people diagnosed with mental illness. While previous studies demonstrated positive outcomes for the use of peer-assisted interventions [41,42] and family involvement [43], their impacts were limited by the need to develop and implement practical methods to engage, screen, and train lay people from the community to support others. Other programs that leverage peer-based support showed positive results in terms of user engagement [44] and efficacy [45] but did not demonstrate ability to empower a large number of peers to enroll and provide support to the extent demonstrated by 7Cups [21].

The approach demonstrated by 7Cups takes into account safety and therapeutic considerations by the way the platform is designed to collect and present listeners’ reputations. Listeners are being continuously evaluated by members for their listening skills and commitment to the 7Cups community, and these evaluations are then presented online for all members to view (in our program only listeners with high evaluation scores were presented in the designated program page). Listeners can also receive a verified listener badge, which means that they were inspected for their listening skills by an experienced listener and passed this inspection. In terms of safety and therapeutic effectiveness, our study was focused on the use of 7Cups as an adjunct to treatment and not as a standalone, which largely affects the safeguards in place. More has to be discussed and examined when it comes to whether there should be other methods for supervision and/or evaluation of actual conversations that occur based on the settings in which such programs are being used.

### Limitations

This study has several limitations. First, while the difference in EPDS scores between 7Cups and TAU was not small (*d*=0.58), it was not significant (*P*=.05). This is not surprising given that the nature of this study was more focused toward evaluation of potential effects, which could be helpful for researchers prior to the initiation of a fully powered examination. Second, the difference in outcomes between the groups is not based on a randomized controlled trial. While the propensity matching paradigm did account for several confounders, there might be other confounders affecting the results. For example, it is possible that there are other factors related to patients enrolling in the study, such as readiness to change, that affected outcomes but could not be balanced (since such factors are not collected in usual care). We believe, however, that the large effect size found for the study group as well as the self-reported effects provide sufficient evidence that could be further investigated using a randomized controlled trial. Third, it might be that study compensation contributed to engagement with the program; however, this is unlikely given that participants were clearly informed that they would receive their compensation regardless of their use of 7Cups and that they would be compensated approximately 4 months after enrollment.

### Conclusions

This study provides data regarding the potential value of 7Cups within the therapeutic process by retrieving user-based empirical data in real-world conditions [46]. Given the development costs of technological resources and the evolving technological landscape, studies that use existing tools rather than developing and evaluating completely new products might be more likely to influence current clinical practices. This study also demonstrated that technology can not only facilitate an unprecedented increase in supportive human resources but also that the quality of emotional support received through this avenue is deemed adequate by people with mental illness. Given that safety controls are adequate, technology could be used across all levels of community, from volunteers, to neighbors, and family members, to provide a novel model of care, where mental illness and mental health needs are not solely addressed behind the closed doors of health care providers.

## Abbreviations

7Cups: 7 Cups of Tea
BAI: Beck Anxiety Inventory
BDI-II: Beck Depression Inventory II
EPDS: Edinburgh Postnatal Depression Scale
PPD: postpartum depression
TAU: treatment as usual
